# Identification of prognostic biomarker of non-small cell lung cancer based on mitochondrial permeability transition-driven necrosis-related genes and determination of anti-tumor effect of ARL14

**DOI:** 10.1186/s41065-025-00379-7

**Published:** 2025-02-03

**Authors:** Zhifei Ma, Wen Chen, Aiping Zhang, Xiaokang Shen, Lin Zheng

**Affiliations:** Department of Thoracic and Cardiovascular Surgery, Nanjing First Hospital, Nanjing Medical University, Nanjing, Jiangsu 210006 China

**Keywords:** Non-small cell lung cancer, Mitochondrial permeability transition-driven necrosis, Biomarker, Prognosis, Immune microenvironment, ARL14

## Abstract

**Background:**

Mitochondrial permeability transition (MPT)-driven necrosis (MPTDN) is a non-apoptotic mode of cell death triggered by oxidative stress and cytosolic Ca^2+^ overload. Recent evidence suggests that activation of MPTND can effectively induce cancer cell death and may represent a novel therapeutic strategy for cancer. Yet, the role of MPTDN-related genes in non-small cell lung cancer (NSCLC) remains unrevealed. This study aimed to identify MPTDN-related biomarkers for predicting prognosis and guiding treatment in NSCLC.

**Methods:**

Gene expression profiles and clinical information of NSCLC were collected from public databases, and MPTDN-related genes were obtained from published article. Differential expressed MPTDN-related genes in NSCLC and control were screened, and molecular clusters were obtained. Based on the differentially expressed genes (DGEs) between clusters, univariate Cox and LASSO regression analyses were performed to screen biomarkers, followed by nomogram construction. Correlations between these biomarkers and immune cell infiltration, immune checkpoints, and chemotherapeutic agents were observed. Expression levels of MPTDN-related biomarkers were detected using RT-qPCR in NSCLC tissues and cells. Moreover, the biological function of ARL14 in NSLCL was verified in vitro.

**Results:**

Thirty-five differential MPTDN-related genes were identified, and two molecular clusters were obtained. Three biomarkers with prognostic values were finally screened, including ARL14, ZDHHC11B, and HLF. Among them, ARL14 was significantly upregulated in tumor samples, while ZDHHC11B and HLF were downregulated. Nomogram containing three genes exhibited predictive accuracy in 1, 3, and 5-year survival rates. Three gene were strongly associated with most immune cells, immune checkpoints, and drugs sensitivity. RT-qPCR confirmed that expression levels of three genes in tissues or cells were consistent with the results of bioinformatics analysis. Finally, ARL14 knockdown inhibited the malignant phenotype of NSCLC cells.

**Conclusion:**

We first performed the comprehensive analysis of MPTDN in NSCLC and screened three NSCLC-related biomarkers as promising biomarkers. ARL14 might be a new potential target for therapy of NSCLC.

**Supplementary Information:**

The online version contains supplementary material available at 10.1186/s41065-025-00379-7.

## Highlights

1. Thirty-five MPTDN-related genes observably dysregulated in NSCLC samples.

2. Two MPTDN-associated molecular clusters with different prognosis were obtained.

3. Three prognostic biomarkers including ARL14, ZDHHC11B, and HLF were screened.

4. Three biomarkers were closely related to prognosis, immune cell infiltration and drug sensitivity.

5. Nomogram based on three biomarkers showed excellent ability for prognosis prediction.

6. Knockdown of ARL14 causes suppression of the malignant phenotype of A549.

**Supplementary Information:**

The online version contains supplementary material available at 10.1186/s41065-025-00379-7.

## Background

Lung cancer is the most common cause of cancer-related death worldwide, and it is estimated that about 340 people die from lung cancer every day, significantly affecting public health [41]. In recent years, great progress has been made in the treatment of lung cancer. In particular, the 3-year relative survival rate for non-small cell lung cancer (NSCLC) increased from 26% in 2004 to 40% in 2017, largely thanks to the emergence of novel therapies [41]. Currently, patients with NSCLC who are eligible for targeted therapy or immunotherapy have longer survival. Depending on the biomarkers, the 5-year survival rate ranges from 15 to 62.5% [36]. Although these types of cancer treatment offer new options for patients, the leadership of the US Food and Drug Administration recognizes that expanding targeted therapies for NSCLC will hold great promise for improving clinical care for cancer patients [27]. Considering that cancer is a disease driven by oncogenic alterations, therapies targeting genes with oncogenic alterations and associated signaling pathways will continue to be an important cancer treatment paradigm [42]. Therefore, the exploration of molecular alterations in NSCLC is helpful to promote the effective treatment of NSCLC.

Over the past decades, various forms of regulated cell death have been explored to develop anticancer drugs [33]. Among them, non-apoptotic cell death has shown great potential in cancer therapy [12]. Mitochondrial permeability transition-driven necrosis (MPTDN) is a non-apoptotic regulatory cell death triggered by specific intracellular perturbations, especially oxidative stress and cytoplasmic Ca^2+^ overload (Galluzzi et al., [10]). The mitochondrial permeability transition pore (MPTP) is a non-selective channel that spans the inner and outer layers of the mitochondrial membrane [3]. Transient MPTP opening participates in the homeostatic regulation of physiological Ca^2+^ and ROS as a release channel, thus protecting mitochondria from oxidative damage; however, its persistent opening inhibits ATP production, causing a massive release of stromal Ca^2+^ and ROS generation, which leads to mitochondrial swelling and release of intermembrane proteins, and ultimately causes cell death [7, 59]. Through continuous research, it has been found that MPTDN can participate in a variety of pathological processes, including cardiovascular diseases, degenerative diseases and cancer [2, 15]. Existing literature has found that the lncRNA signatures identified based on MPTDN can be used as diagnostic or prognostic biomarkers for several cancers, such as cervical cancer and renal clear cell carcinoma [12, 14]. Notably, recent evidences reveals that the isobavachalcone can exert anti-cancer role by inducing Ca^2+^ overload in breast cancer cells to promote the formation of MPT and trigger ROS-mediated MPTDN [50]. Similarly, cannabinoids also reverse glucocorticoid resistance in leukemia via triggering this necrotic process [29]. Therefore, investigating the molecular mechanisms of MPTDN can provide new insights for the cancer treatment. However, studies on MPTDN in NSCLC are still scarce [50].

Nowadays, there is a growing tendency to integrate molecular biological information into prognostic models, thereby improving the accuracy of tumor prognosis prediction [4]. This integration can also provide clinicians with prognostic information for patients, which can help to develop personalized treatment strategies and enhance clinical outcomes [25]. Moreover, identification of molecular subtypes can assist in the precise classification of tumors and guide therapy [37]. For example, lung adenocarcinoma patients the classification of into three subtypes based on ferroptosis-related genes, and they show different prognostic pattern, which is important for distinguishing the effect of immunotherapy [56]. In addition, Zhu et al. used necroptosis-related genes to classify NSCLC patients into two different subtypes, providing potential value in guiding chemotherapy [58]. Despite the increasing number of studies providing insights into molecular subtypes, the prognosis prediction of NSCLC patients remains suboptimal. Thus, more factors related to prognosis need to be considered.

In view of the above limitations, we systematically explored the relationship between MPTDN-related genes and NSCLC. Based on the expression of MPTDN-related genes, MPTDN-associated molecular subtypes and expression patterns were identified. Afterwards, using key prognostic biomarkers, a novel prognostic model was developed to provide more accurate and individualized prognostic prediction for patients with NSCLC. This study helps to deepen the understanding of MPTDN characteristics and provide new ideas for optimizing the clinical management and treatment of NSCLC.

## Methods

### Data source

Gene expression data and corresponding clinical phenotype data of LUAD and LUSC samples in The Cancer Genome Atlas (TCGA) database were downloaded from UCSC Xene (https://xenabrowser.net). This TCGA cohort contained 1013 NSCLC tumor tissue and 109 control samples, of which 993 tumor samples had detailed survival and clinical information. Microarray dataset GSE30219 was downloaded from the gene expression omnibus (GEO) database, which comprises of 272 NSCLC samples and 14 control samples. Among the 272 tumor samples, 268 samples had detailed survival information. GEO30219 dataset was used as an external independent validation dataset.

### Differentially expressed MPTDN-related genes

A total of 39 MPTDN-related genes was retrieved from a previous study [24], and expression data of these genes were extracted from TCGA cohort. Differentially expressed MPTDN-related genes between tumor and control samples were screened using Wilcox test, which was then visualized using ggplot2 package.

### Consensus clustering analysis

There is growing evidence that clustering analysis achieved by ConsensusClusterPlus can discover molecular subtypes with different gene expression patterns, which is of great significance for understanding the molecular mechanism of cancer and guiding personalized treatment [22, 23, 30]. Based on the expression data of the differentially expressed MPTDN-related genes, Consensus clustering analysis was conducted to classify lung cancer samples into different molecular subtypes using ConsensusClusterPlus package. Results of cluster analysis was evaluated by principal component analysis (PCA). Kaplan-Meier (K-M) survival analysis was conducted using survival package to compare the survival of patients in two molecular subtypes. Clinical characteristics of samples in two molecular subtypes were also compared using chi-square test.

### Differential expression analysis

Differential expression analysis between tumor and control groups, as well as between two molecular subtypes were conducted using limma package, followed by Benjamini & Hochberg correction for multiple tests. Differentially expressed genes (DEGs) of these two comparison groups were screened with cut-off values of adjusted *P* < 0.05 and |log_2_fold change (FC)| >1. The overlapped genes between two sets of DEGs were screened using VennDiagram package.

### Functional enrichment analysis and protein-protein interaction (PPI) construction

To explore the biological function of genes, enrichment analysis of gene ontology (GO) terms and Kyoto Encyclopedia of Genes and Genomes (KEGG) pathways were performed using clusterprofiler package. GO terms included three directions, namely biological process (BP), molecular function (MF), and cellular component (CC), which can fully reveal the specific functions of genes [1]. In addition, KEGG pathway is commonly used to elucidate the role of genes in various metabolic and signaling pathways [17]. Interactions among protein encoded genes were predicted based on STRING database (version 11.0, http://string-db.org/) with the required minimum interaction score set as “Medium confidence (0.40)”. The obtained PPIs were visualized into a PPI network using Cytoscape software.

### Identification of prognostic biomarkers

Univariate Cox regression analysis was performed to screen genes that significantly associated with survival of patients, using *P* < 0.05 as cut-off value. The optimal prognostic signature genes were further identified by LASSO regression with 20-fold cross validation (family =“cox”, nfold = 20) using the glmnet package (version 4.0–2). Next, survival analysis of these prognostic biomarkers was conducted. Briefly, lung cancer samples were classified into high and low gene expression groups based on the optimal cut-point determined by survminer package, and differences on survival of patients between high and low expression groups were analyzed by K-M survival analysis. Differences on expression of these prognostic biomarkers in tumor and control samples were explored using wilcox test.

### Development of nomogram predictive model

For easy of clinical use, the identified prognostic biomarkers were included to develop a nomogram model using rms package to predict 1-, 3- and 5-year survival of lung cancer patients. Calibration, decision curve analysis (DCA) and clinical impact curves (CIC) were plotted to evaluate the performance of the nomogram model.

### Single-gene gene set enrichment analysis (GSEA)

To investigate the potential function of prognostic biomarkers, single-gene GSEA based on GO biological process and KEGG gene sets was conducted using clusterProfiler package. Benjamini & Hochberg correction for multiple tests was performed, and adjusted *P* value < 0.05 was used as threshold.

### Construction of regulatory network

NetworkAnalyst database (https://www.networkanalyst.ca/) was used to explore the miRNAs that could target these prognostic biomarkers, with parameters set as: Specify organism: human; Gene-miRNA interaction database: TarBase v8.0. The obtained miRNA-mRNA regulatory pairs were visualized using Cytoscape software.

### Evaluation of immune status

The infiltration fraction of 28 types of immune cells in sample was analyzed using ssGSEA algorithm. Differences on immune cell infiltration between control and tumor samples were compared using wilcox test. The correlations between biomarkers and differential immune cells were assessed by Spearman. Besides, expression of 44 immune checkpoints between tumor and control samples were compared using wilcox test.

### Prediction of chemotherapeutic drugs

Based on the expression profile data of cell line provided in GDSC database (https://www.cancerrxgene.org/) and the gene expression profile of lung cancer patients in TCGA, Ridge regression model was constructed using pRRophetic package to predict IC50 value of each drug. The Spearman correlation coefficient between chemotherapeutic drugs and biomarkers, and the correlations scatterplot was plotted using ggplot2 package.

### Clinical sample collection

Tissue samples were collected from nine pairs of NSCLC and adjacent normal tissues that under surgery at Nanjing First Hospital. Samples were evaluated by pathologists. This research was authorized by the ethics committee of the hospital (Approval No. KY20190404-03-KS-01), and the written informed consents were acquired from all patients.

### Cell culture

One human bronchial epithelial cell line (BEAS-2B) and two NSCLC cell lines (A549 and NCI-H1299) were purchased from the Cell Resource Center of the Chinese Academy of Sciences (Shanghai, China). A549 and BEAS-2B were maintained in DMDM medium, while NCI-H1299 were cultured in RPMI-1640 medium. These mediums were supplemented with 10% FBS and 1% penicillin-streptomycin solution. All cell lines were cultured in an incubator at 37 °C with 5% CO_2_.

### Construction of ARL14 knockdown cell lines

A549 cells in logarithmic phase were collected and seeded in 24-well plates at a density of 4 × 10^4^ cells/well. Transfection was performed when the cells grew to 80–90%. Three different ARL14 siRNAs (sequences are listed in Table 1) and their control (si-NC) were designed and synthesized by Generay Biotechnology (Shanghai, China). According to the manufacturer’s instructions, NC, ARL14-siR-1, ARL14-siR-2, and ARL14-siR-3 sequences were transfected into cells using Lipofectamin 2000 (11668-027, Invitrogen, USA). After standing at 37 °C for 6 h, complete medium was added and incubated for 48 h, followed by collection of cells for subsequent experiments. The knockdown efficiency of ARL14 was detected by using RT-qPCR.

**Table 1:** Sequences of primer used in this study

Primer name	Sequence (5’−3’)
ARL14-F	AAATCCGCAAACCAAACAAG
ARL14-R	TTCCAACTCGATCATTTCCACAT
ZDHHC11B-F	GCCATCCTGCTGTATGTCCT
ZDHHC11B-R	GGCCTTCAGGTAGATGTGGA
HLF-F	CTGGGGCCTACCTTATGGGA
HLF-R	GGGGAATGCCATTTTCTGACA
si-ARL14-1-S	GCUUGCUAAGGAUAUUACCAC
si-ARL14-1-AS	GGUAAUAUCCUUAGCAAGCUU
si-ARL14-2-S	AUGUGCCUGUUGUUCUAUUAGTT
si-ARL14-2-AS	CUAAUAGAACAACAGGCACAUTT
si-ARL14-3-S	UAGUAAGCUGUCUGCUAAUAGTT
si-ARL14-3-AS	CUAUUAGCAGACAGCUUACUATT
GAPDH-F	TGACAACTTTGGTATCGTGGAAGG
GAPDH-R	AGGCAGGGATGATGTTCTGGAGAG

*F* forward, *R *reverse, *S *sense, *AS *antisense

### RT-qPCR assay validation

Total RNA from the tissues and cells was extracted using the TransZol Up kit (Cat. ET111-01, TransGen Biotech, Beijing, China), followed by determination of RNA purity and concentration via spectrophotometry and 1% agarose. After reverse transcription using a cDNA synthesis kit (Cat. AU341-02, TransGen Biotech), qPCR was performed with a PerfectStart^®^ Green qPCR kit (Cat. AQ601-02, TransGen Biotech). This assay was completed on a fluorescence quantitative PCR instrument (No. Q2000B, LongGene, Hangzhou, China). The relative gene expression was calculated using the 2^−△△CT^ method, with GAPDH as the internal reference gene. The sequences of primers are listed in Table 1.

### Cell viability assay

Cell viability was assayed using a commercial CCK-8 kit (C0038, Beyotime, Shanghai, China). After transfection, cells were inoculated in 96-well plates (1 × 10^4^ cells/well) and incubated overnight at 37 °C, 5% CO_2_. Then, each well was treated with CCK-8 reagent (10 µL) for 60 min according to the manufacturer’s procedure. Finally, the optical density at 450 nm was measured using a microplate reader to represent cell viability.

### Colony formation assay

The transfected cells were seeded in 6-well plates (400 cells/well) and then incubated in a 5% CO_2_ incubator at 37 °C for 14 d. After washing twice with PBS, the plates were fixed with 4% paraformaldehyde for 10 min and stained with crystal violet for 10 min. Finally, the number of clone cells was counted under a light microscope.

### Cell migration and invasion assays

Migration and invasion experiments were conducted using transwell. For migration assay, transfected cells were resuspended in serum-free medium (3 mL) and inoculated into the upper chamber of the transwell at a density of 2 × 10^4^/well. Unlike, the assessment of invasion required pre-coating of Matrigel in the upper chamber. In two experiments, 20% PBS medium was supplemented to the lower chamber of the transwell. After 48 h of incubation in a cell culture incubator (37 °C, 5% CO_2_), the transwell chambers were removed and fixed with 4% paraformaldehyde (500 µL) for 20 min, followed by staining with crystal violet for 20 min. Unmigrated or uninvaded cells were gently wiped with a cotton swab and then cells were counted under a microscope.

### Cell apoptosis assay

The Annexin V-FITC/PI kit (556420, BD Biosciences, Jiangsu, China) was utilized to assess cell apoptosis. Stably transfected cells were collected and digested with trypsin, followed by centrifugation at 1000 rpm for 5 min. After resuspension by PBS, cells were stained by incubation with Annexin V-647 (5 µL)/PI (5 µL) for 20 min at room temperature (20–25 °C) in the dark, and then detected by flow cytometry.

### Statistical analyses

Data analyses were conducted using R language (Version 3.6) or Graphpad prism (Version 5.0). Univariate and LASSO Cox regression analyses were used to screen biomarkers with prognostic significance. KM method was employed for survival analysis, and Spearman was used to analyze the correlation between genes and immune cells, immune checkpoints, as well as drugs. Each experiment was repeated three times and data were presented as mean ± standard deviation. Comparisons between the groups were examined using the wilcox test (two groups) or one-way ANOVA (multiple groups). *P* value < 0.05 was defined as statistical significance.

## Results

### Identification of two molecular clusters based on MPTDN-related genes

**Fig. 1:**
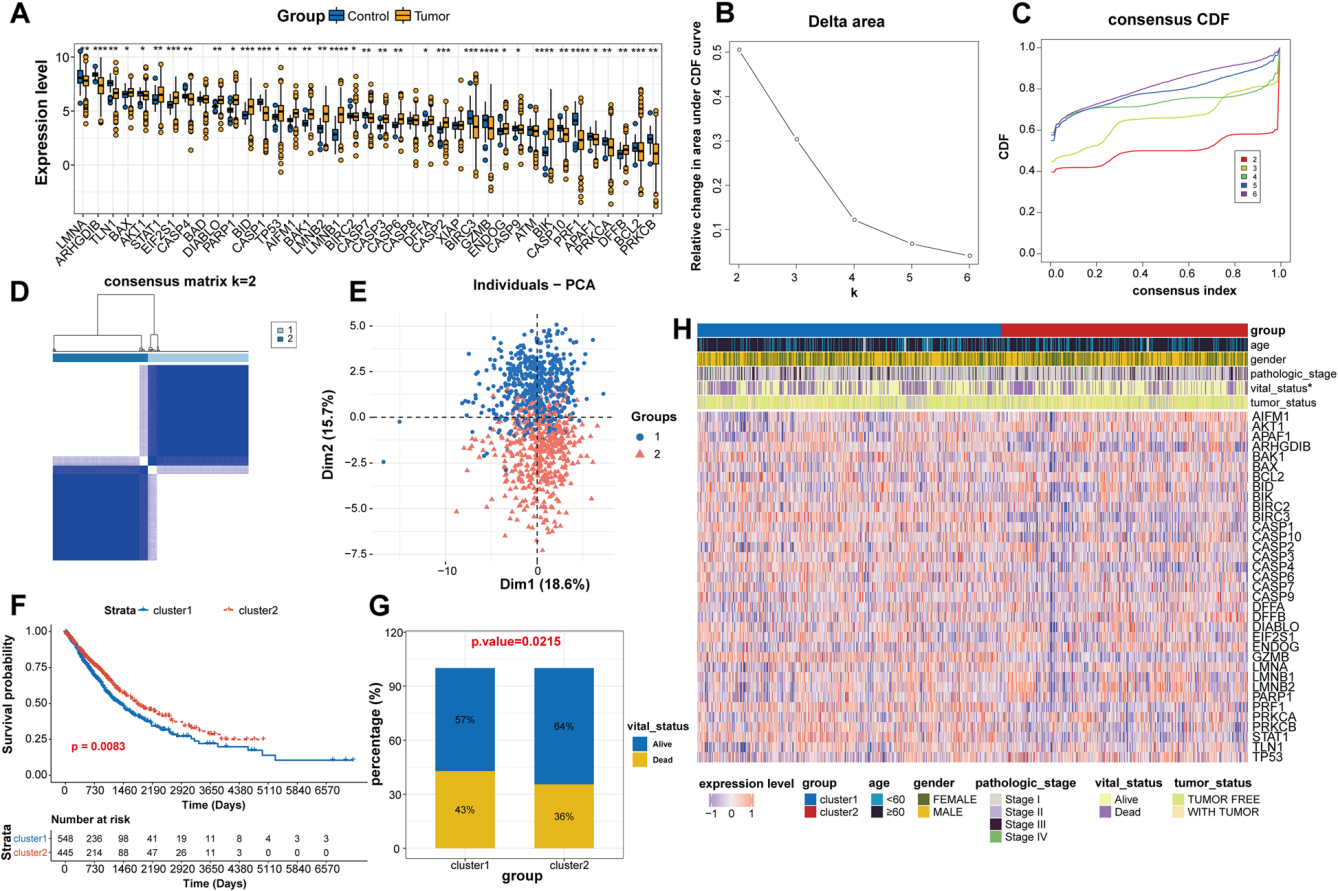
Identification of MPTDN-associated molecular subtypes via consensus clustering. **A** Expression levels of 39 MPTDN-related genes between control and tumor samples. **B** and **C** Delta area curves for consensus clustering represent the relative change in area under the cumulative distribution function (CDF) curve for k = 2 to 6. **D** Heatmap consensus matrix show K = 2 as the optimal clustering result. **E **PCA plot shows that cluster 1 and cluster 2 samples can be clearly distinguished. **F** KM curve analysis reveals the significant differences in overall survival of the two clusters. **G** Histogram shows the differences in vital status between cluster 1 and cluster 2. **H** Heatmap shows the relationship between gene expression levels and clinical features. *P < 0.05; **P < 0.01; ***P < 0.001

Among the 39 MPTDN-related genes retrieved from a previous study [24], 35 genes displayed significant differences between NSCLC and control samples (Fig. 1A). Based on these genes, ConsensusClusterPlus was employed for consistent cluster analysis. K = 2 was found to be the optimal clustering result from the CDF curve, the relative change of the area under curve, and the matrix diagram, indicating that the patients could be clearly categorized into two clusters (Fig. 1B and D). PCA plot showed that the two cluster samples were distinctly separated (Fig. 1E). Importantly, patients in cluster 2 had a significantly survival advantage over those in cluster 1 (*P* = 0.0083, Fig. 1F). We also observed a significant association between vital status and clusters (*P* = 0.0215, Fig. 1G). As expected, more alive samples were observed in cluster 2 compared to cluster 1. Meanwhile, the heatmap revealed the specific connection between clinical features and 35 genes in different clusters (Fig. 1H). These genes displayed different expression patterns in the two clusters. For example, compared with cluster 1, ARHGDIB, BID, GZMB and PRF1 were clearly down-regulated in cluster 2, whereas AIFM1, APAF1 and ENDOG were up-regulated.

### Screening of DEGs from two clusters as well as TCGA cohort

**Fig. 2:**
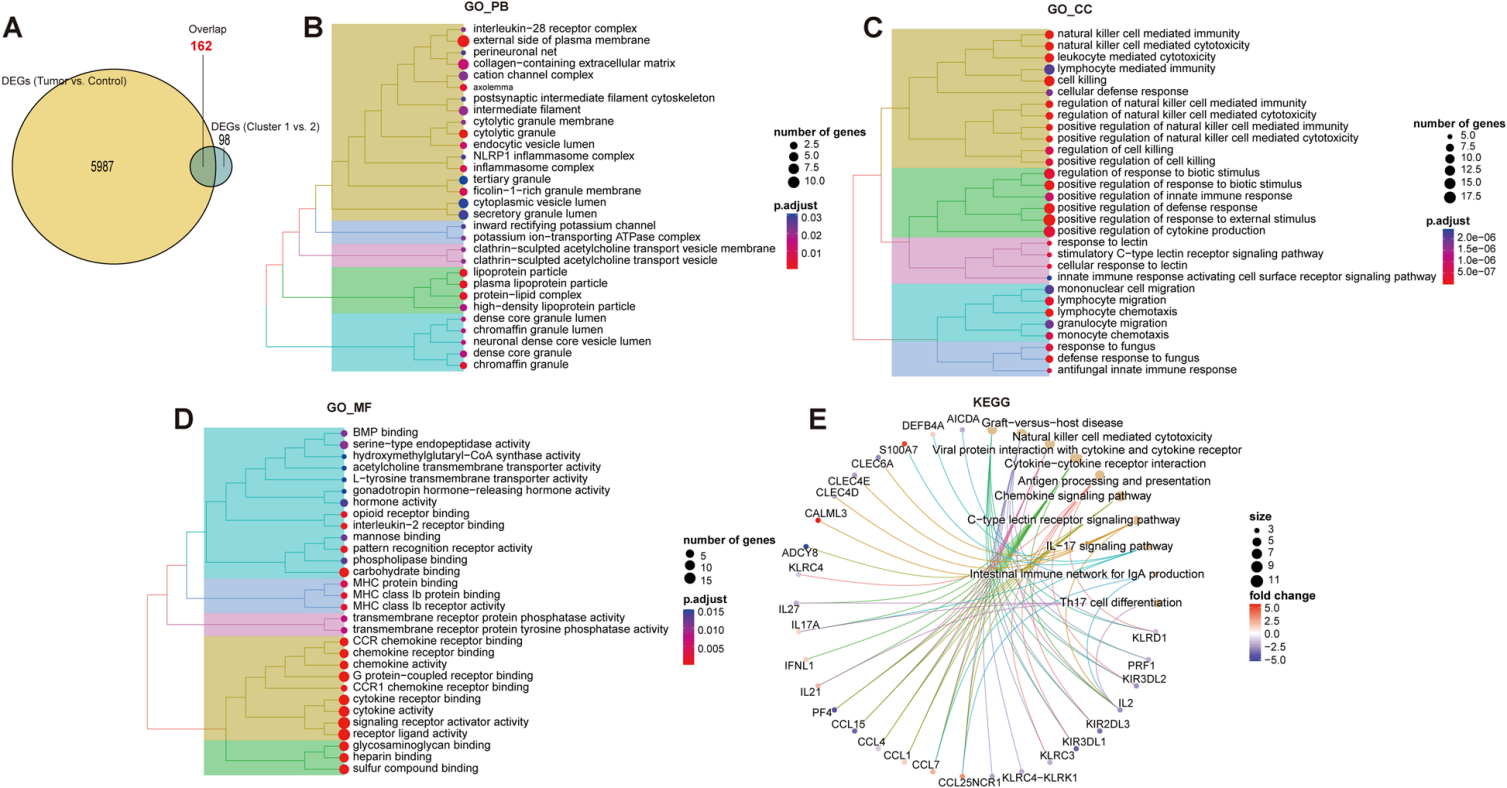
Screening of DEGs and functional enrichment analysis. **A** Venn diagram shows the integration of DEGs in cluster 1 vs. cluster 2 and tumor vs. control. GO_BP (**B**), GO_CC (**C**), GO_M (**D**), and KEGG pathway (**E**) analyses of 162 overlapped genes

According the thresholds set in methods, 260 DEGs were identified within cluster 1 vs. cluster 2. Besides, 6149 DEGs were obtained between NSCLC tumor and control samples from TCGA dataset. Volcano plot and heatmap was used to visualize these DEGs, and it was found that DEGs could distinguish the two groups of samples (Figure S1A and S1B). Following, VennDiagram was utilized to integrate the two sets of DEGs, and 162 overlapped genes were obtained for further analyses (Fig. 2A). These genes may simultaneously play key roles in different MPTDN-associated subtypes as well as NSCLC. To explore the biological function involved in these genes, functional enrichment analysis was performed using clusterProfiler. Results showed that these DEGs were obviously enriched in 511 GO_BP, 41 GO_CC, 81 GO_MF, and 26 KEGG pathways. Results of top 30 GO terms and top 10 pathways were displayed. In brief, BP analysis revealed that genes were mainly enriched in natural killer mediated cytotoxicity-related functions, such as cell killing and natural killer cell mediated immunity (Fig. 2B). CC analysis showed genes were mainly enriched in cytolytic inflammasome intermediate filament-related terms, such as cytolytic granule and external side of plasma membrane (Fig. 2C). MF analysis indicated genes were enriched in cytokine CCR chemokine activator-related functions, including cytokine activity and receptor ligand activity (Fig. 2D). Moreover, top three KEGG pathways were graft-versus-hot disease, natural killer cell mediated cytotoxicity, and viral protein interaction with cytokine and cytokine receptor (Fig. 2E). In addition, PPI network revealed the interactions of 162 overlapped genes, including 173 edges (Figure S2).

### Identification of three prognostic biomarkers via univariate cox and LASSO analyses

**Fig. 3:**
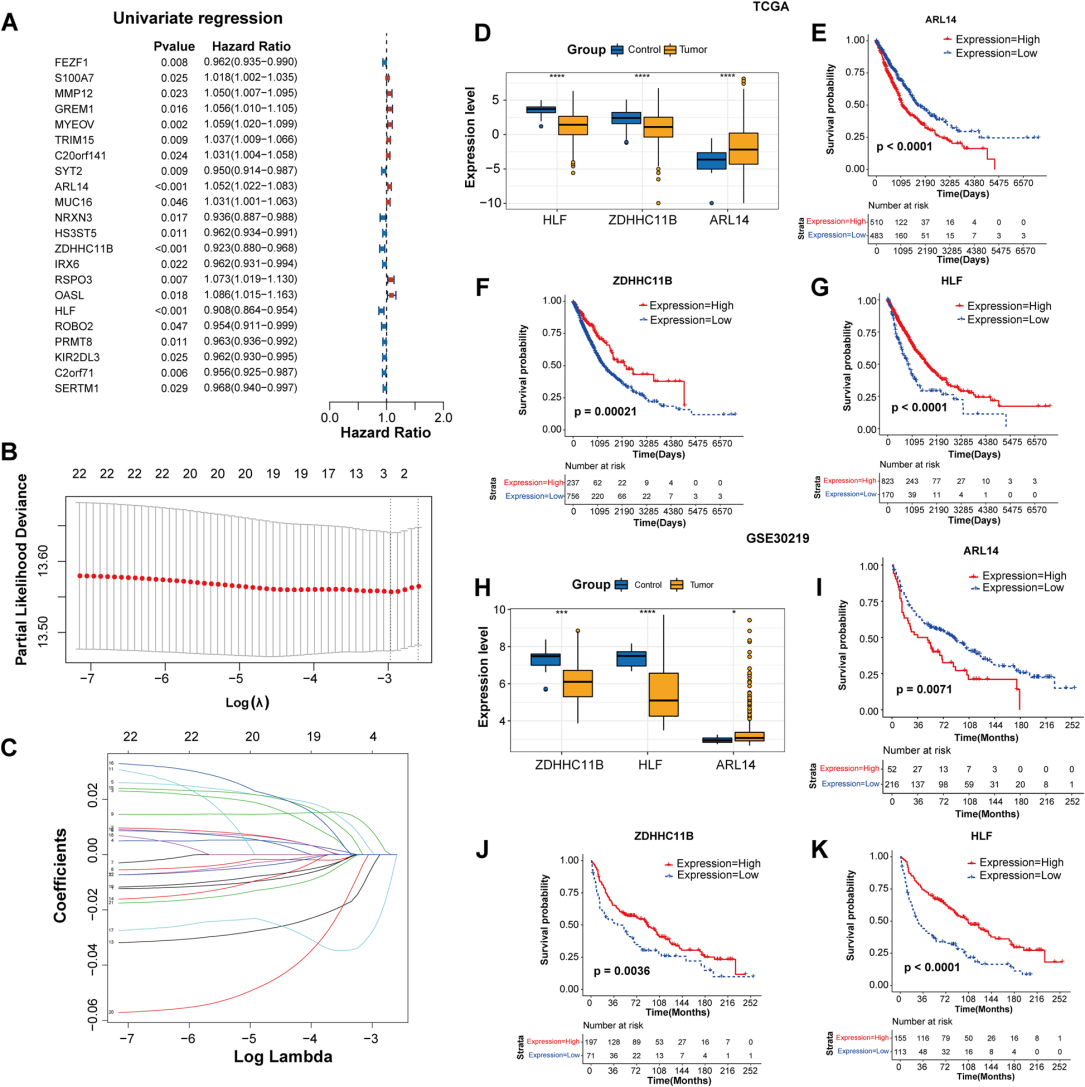
Identification of prognostic biomarkers via univariate Cox and LASSO regression analyses. **A** Forest plot of prognostic-related genes detected by univariate Cox analysis. **B** Partial likelihood deviance on the prognostic biomarkers. **C** LASSO coefficient profiles on the prognostic biomarkers. **D** Expression levels of three genes in control and tumor samples from TCGA. KM curves analysis of ARL14 (**E**), ZDHHC11B (**F**), and HLF (**G**) in TCGA cohort. **H** Expression levels of three genes in control and tumor samples from GSE30219. KM curves analysis of ARL14 (**I**), ZDHHC11B (**J**), and HLF (**K**) in GSE30219 cohort. *P < 0.05; **P < 0.01; ***P < 0.001; ****P < 0.0001

To determine the clinical application value of 162 genes, univariate and LASSO regression analyses were conducted. Based on the training set (TCGA cohort), univariate Cox regression analysis identified 22 genes associated with overall survival (Fig. 3A). Next, LASSO regression analysis was conducted to further narrow down the candidate genes. Results showed that lambda = 0.0514 corresponded to the optimal model, so three genes were finally selected as prognostic biomarkers, namely, ARL14, ZDHHC11B, and HLF (Fig. 3B and C).

To analyze the prognostic value of the three genes, expression level and KM analysis were performed in two datasets. In training set (TCGA), ZDHHC11B and HLF were significantly downregulated in tumor samples, whereas ARL14 was significantly upregulated (*P* < 0.05, Fig. 3D). There was a significant difference in survival between low- and high-level of gene expression. Specifically, patients with high expression of ZDHHC11B and HLF were accompanied by better survival times, while those with high expression of ARL14 had worse prognosis (Fig. 3E and G). The same results were also observed in the validation set (GSE30219). In brief, the expression of the three genes displayed difference between tumor and control samples, and their expression levels were strongly correlated with the prognosis of patients (Fig. 3H and K). Notably, previous studies confirmed that increased ARL14 expression was strongly associated with poor survival of NSCLC patients, while the decreased ZDHHC11B and HLF expression predicted poor survival of NSCLC patients [5, 47, 55]. These findings further support the prognostic value of these three genes in NSCLC.

### Establishment of nomogram model for predicting survival probability

**Fig. 4:**
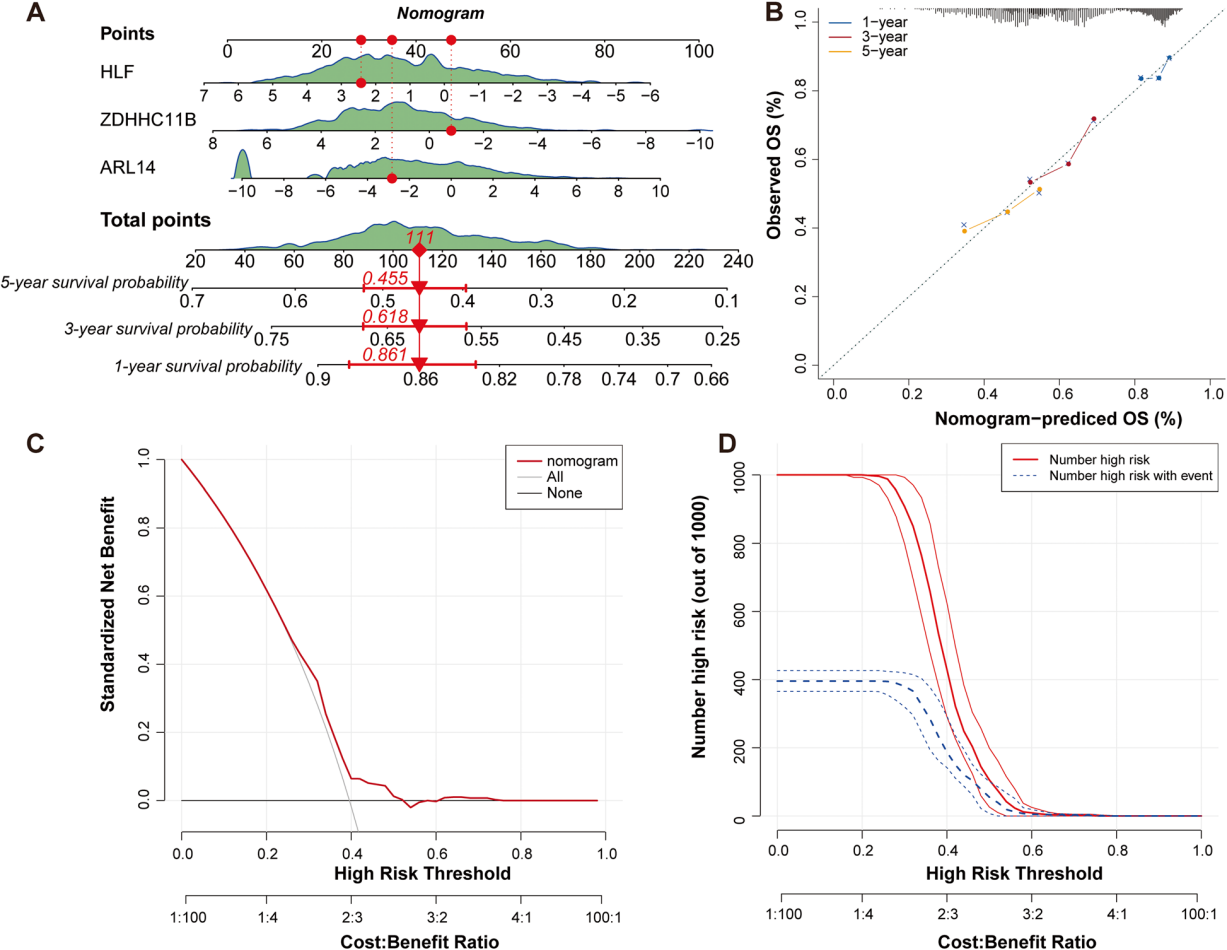
Establishment of nomogram model based on three prognostic biomarkers. **A** Nomogram containing three biomarkers for predicting 1, 3, and 5-year survival rates. **B** Calibration curves of the nomogram for the 1, 3, and 5-year overall survival. **C** DCA curve for evaluating the clinical application value of the nomogram. **D** CIC for nomogram model

Following, a nomogram was created to predict the probability of survival in NSCLC patients based on three genes (Fig. 4A). A value was assigned to each factor and the values of the three factors were summed to generate a total score. This score was utilized to predict patient survival at 1-, 3-, and 5-year, with higher scores indicating poorer survival. Then, the performance of nomogram model was evaluated. Calibration curves showed that the predicted survival of patients at 1, 3, and 5 years was consistent with the actual results (Fig. 4B). DCA revealed that the nomogram had clinical benefit within the high-risk threshold range of 0–1 (Fig. 4C). In addition, CIC further confirmed the accurate predictive ability of the model under the high-risk threshold (Fig. 4D). Overall, these results suggest that the nomogram had excellent predictive capability.

### GSVA revealed the specific signaling mechanisms of ARL14, ZDHHC11B, and HLF

**Fig. 5:**
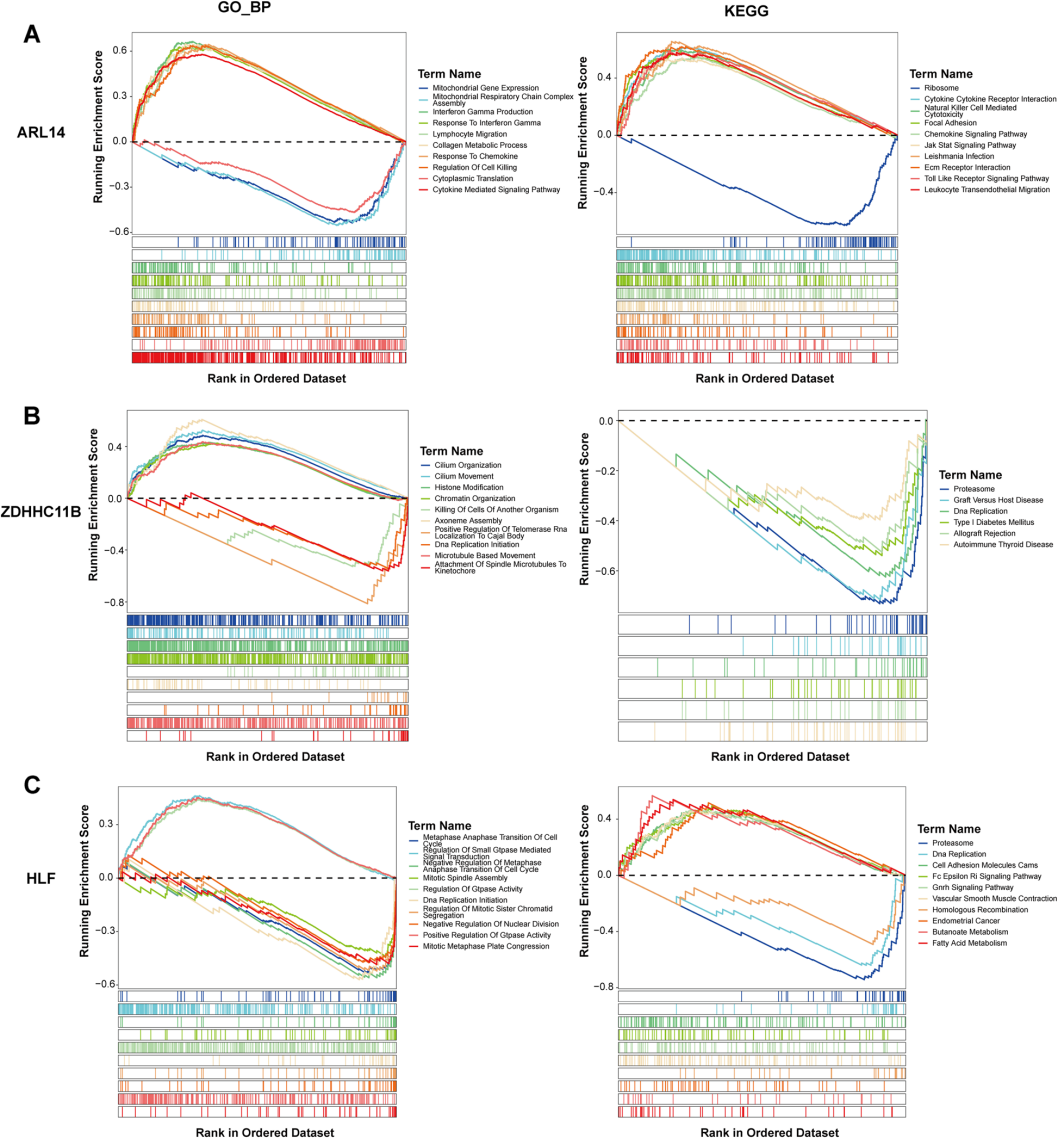
GSVA results of GO_BP and KEGG pathways for ARL14 (**A**), ZDHHC11B (**B**), and HLF (**C**)

We employed GSEA to explore the specific biological functions and KEGG pathways involved in these three genes. High expression of ARL14 was mainly enriched in interferon gamma production (GO_BP), JAK/STAT signaling pathway and toll like receptor signaling pathway (KEGG pathway). Low expression of ZDHHC11B was primarily associated with positive regulation of telomerase RNA location to Cajal body (GO_BP) and proteasome (KEGG pathway). As for HLF, its low expression was significantly enriched in DNA replication initiation (GO_BP) and proteasome (KEGG pathway). Results of the top 10 items in GSEA are detailed in Fig. 5A and C.

### Establishment of mRNA-miRNA regulatory network

A total of 265 miRNA-mRNA pairs were obtained and then visualized using Cytoscape software (Figure S3). Results indicated that both HLF and ARL14 were regulated by hsa-miR-124-3p, hsa-miR-212-3p, and hsa-miR-941.

### Association between three prognostic biomarkers and immunity characteristics

**Fig. 6:**
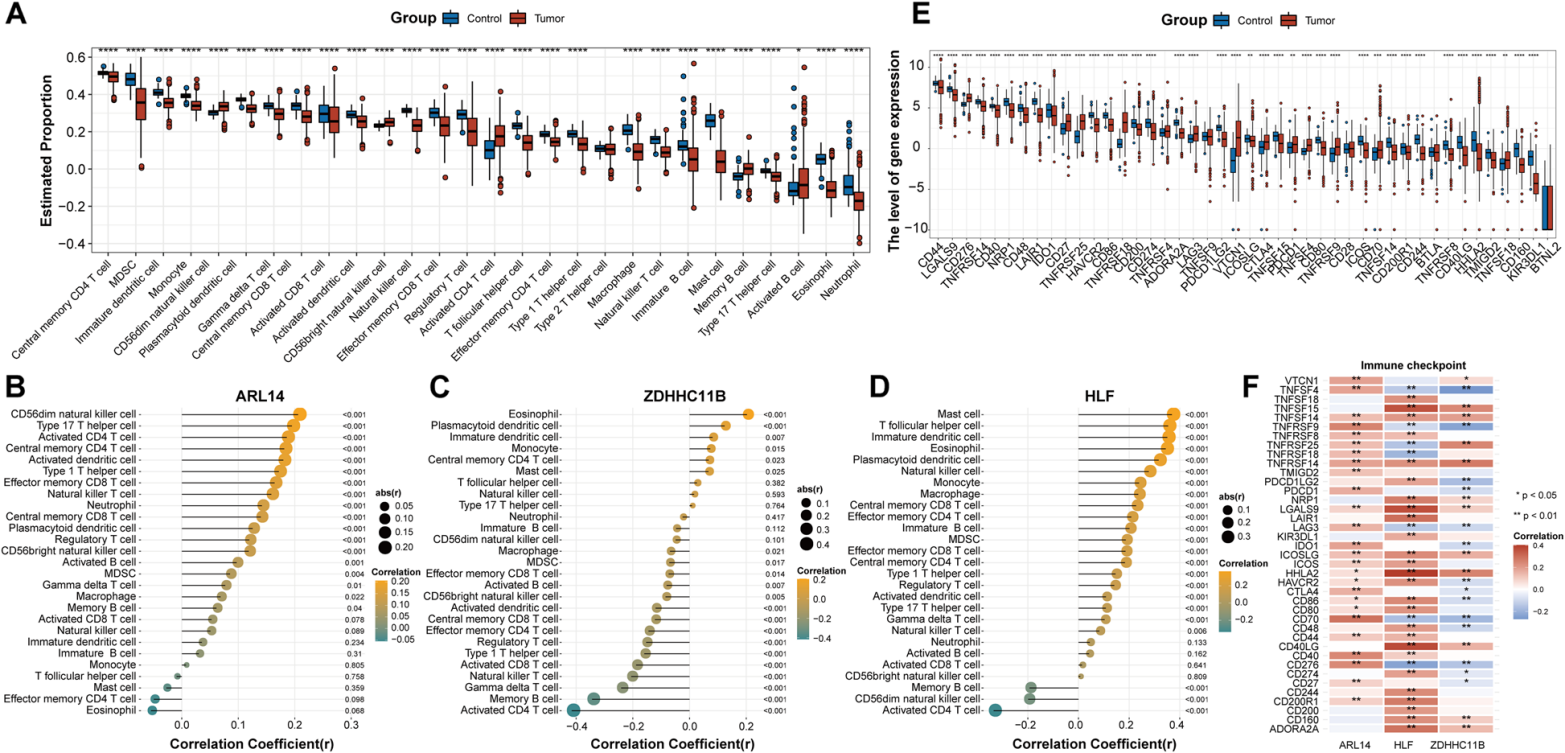
Correlation analysis of biomarkers and immune cells as well as immune checkpoints. **A** Differential analysis of immune cell infiltration between the control and tumor samples. **B**-**D** Correlation of ARL14, ZDHHC11B, and HLF with immune cells. **E** Differentially expressed analysis of immune checkpoints between the control and tumor samples. **F** Correlation heatmap of three biomarkers and immune checkpoints. *P < 0.05; **P < 0.01; ***P < 0.001; ****P < 0.0001

Considering the immune infiltrating cells serves a crucial role in the tumor microenvironment, we applied ssGSEA algorithm to analyze the correlation between immune cells and three gene expression levels. A total of 27 out of 28 immune cells displayed significant differences between tumor and control samples (Fig. 6A). Low infiltration of most immune cells was observed in the NSCLC group. ARL14 was strongly positively correlated with CD56dim natural killer cell and type 17 T helper cell (Fig. 6B). ZDHHC11B showed significantly positive correlation with eosinophil and plasmacytoid dendritic cell, while negative correlation with activated CD4 T cell and memory B cell (Fig. 6C). HLF was positively related to mast cell and T follicular helper cell, while negatively related to activated CD4 T cell and CD56dim natural killer cell (Fig. 6D). Furthermore, the relationship between three biomarkers and immune checkpoint was analyzed. Thirty-three immune checkpoints showed statistically significant differences between control and cancer samples (Fig. 6E). There were significant correlations between the three genes and most of the immune checkpoints (Fig. 6F). Among them, HLF strongest positively associated with HHLA2.

### Association between three gene expressions and drug sensitivity

**Fig. 7:**
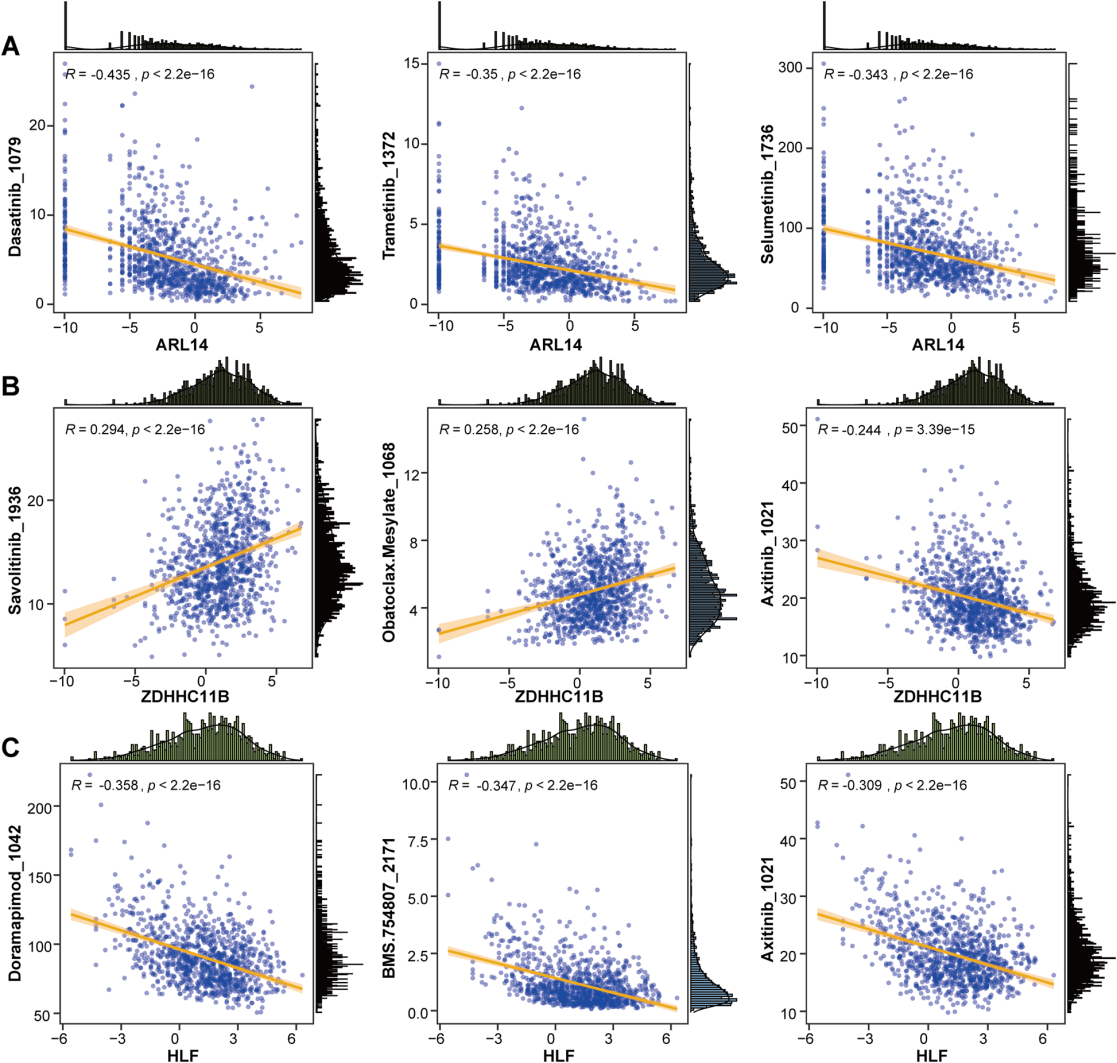
Association of prognostic biomarkers and drug sensitivity. **A** ARL14. **B** ZDHHC11B. **C** HLF

To explore the potential value of three prognostic biomarkers in NSCLC chemotherapy, a correlation analysis of gene expression level and drugs was performed. Here, we displayed the top three drugs (ranked by p value) associated with each gene. Specifically, ARL14 was negatively correlated with the sensitivity of dasatinib_1079, trametinib_1372, and selumetinib_1736 (Fig. 7A). ZDHHC11B was positively associated with the sensitivity of savolitinib_1936 and obatoclax.mesylate_1068, whereas negatively associated with axitinib_1021 (Fig. 7B). HLF exhibited a significant negative correlation with doramapimod_1042, BMS.754807_2171, and axitinib_1021 (Fig. 7C).

### Expression level of three biomarkers in tissue samples or NSCLC cells detected by RT-qPCR

**Fig. 8:**
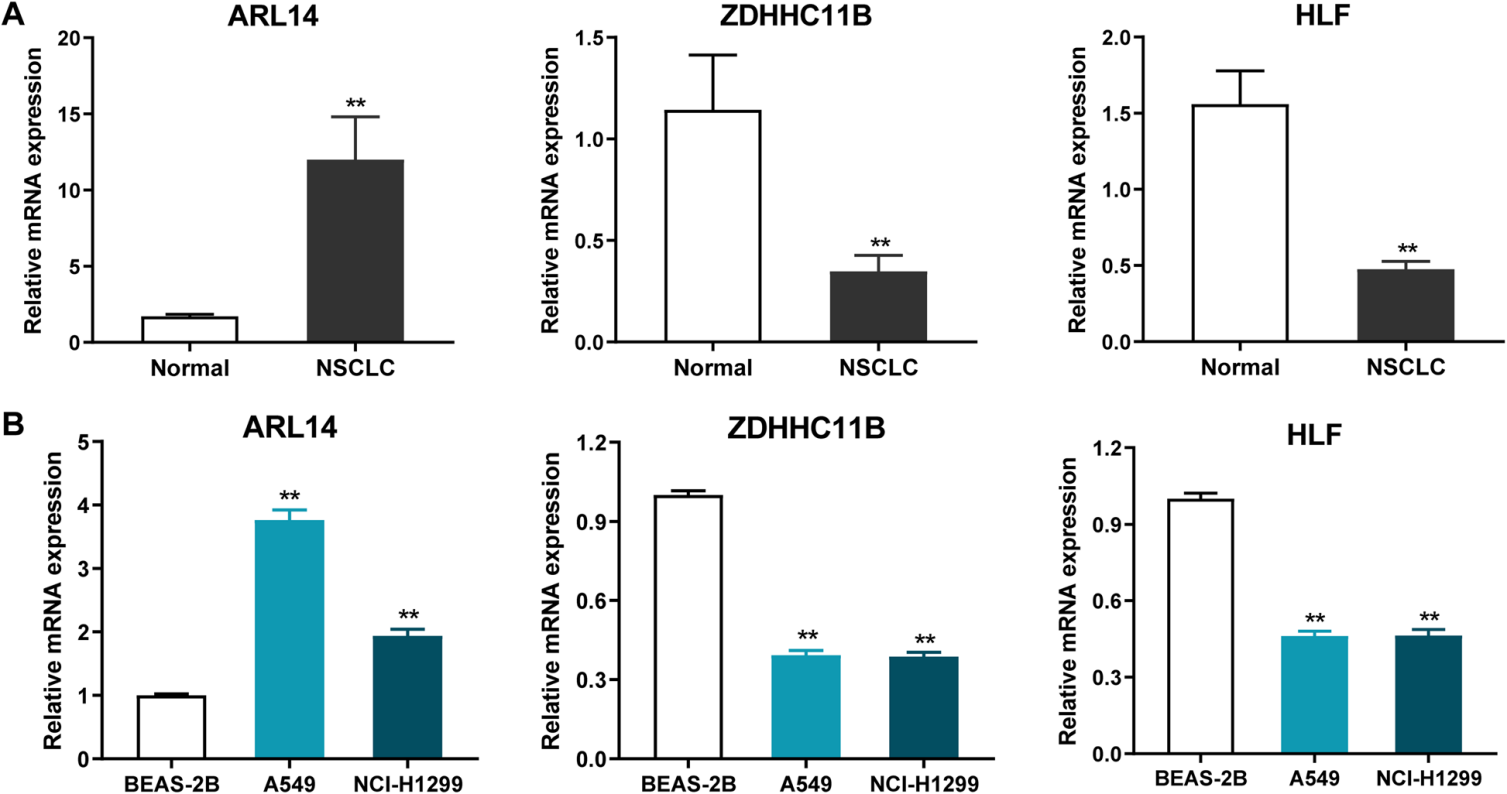
RT-qPCR results of three prognostic biomarkers expression levels in the normal and NSCLC tissues (**A**) and cells (**B**). **A** ARL14. **B** ZDHHC11B. **C** HLF. **P < 0.01

To verify of the accuracy of the bioinformatics analysis, RT-qPCR was utilized to examine the expression levels of three biomarkers in the NSCLC samples and cells. As expected, ARL14 was significantly up-regulated in tumor samples, while ZDHHC11B and HLF were clearly down-regulated in tumor (Fig. 8A). The same results were also observed in vitro. Briefly, compared with BEAS-2B cells, both A549 and NCI-H1299 cells exhibited elevated ARL14 expression as well as suppressed ZDHHC11B and HLF expression (Fig. 8B). Considering that ARL14 was a risk factor for NSLCL and may act as an oncogene. Therefore, we subsequently explored the biological functions of ARL14 in NSCLC. Among two NSCLC cell lines, the A549 cell line with higher expression of ARL14 was selected as the research object.

### ARL14 knockdown suppresses the malignant phenotype of NSCLC cells

**Fig. 9:**
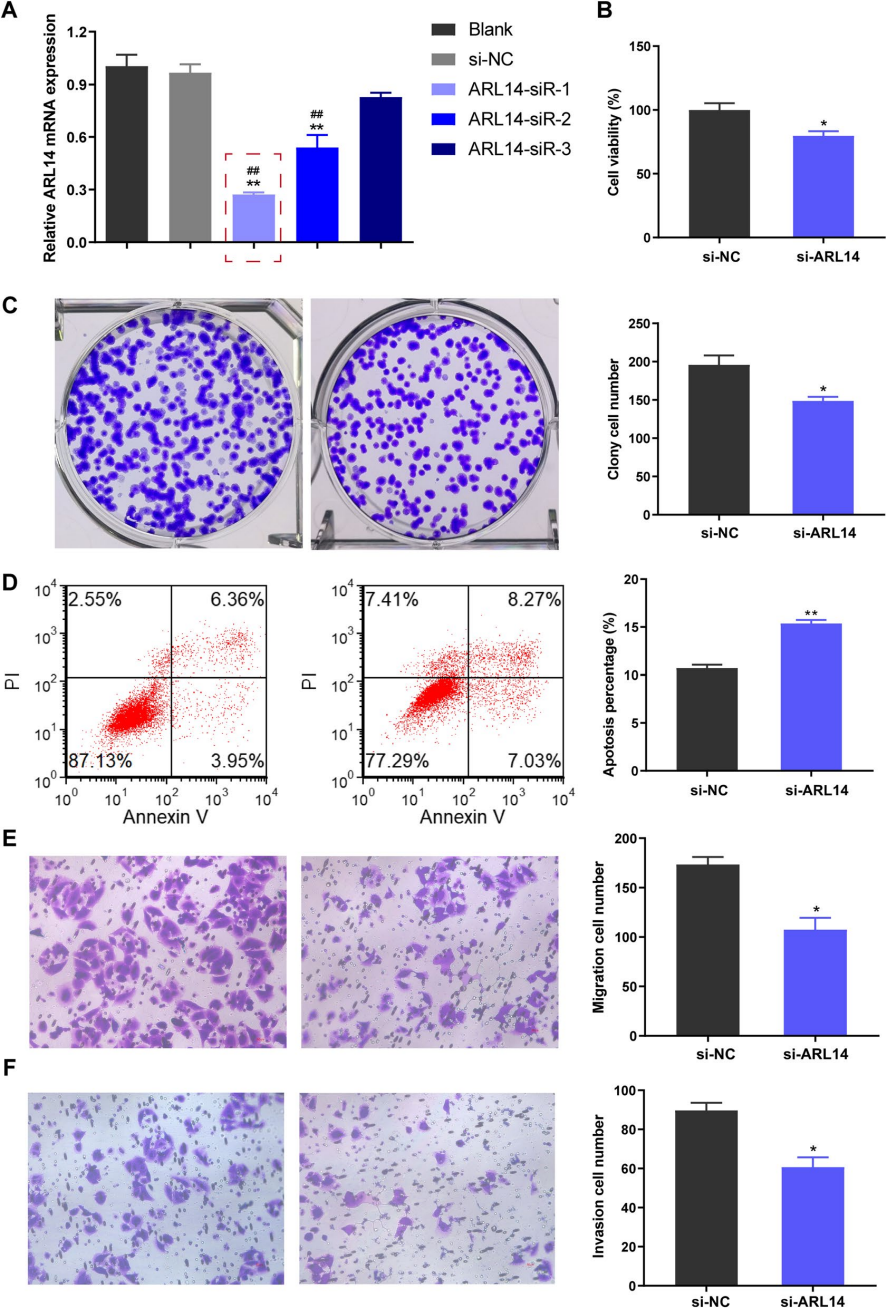
ARL14 knockdown inhibited the malignant phenotype of NSCLC cells. **A** qRT-PCR assay showed the mRNA level of ARL14 in A549 cells after transfection of three diferent siRNAs. **B** Cell viability of A549 was assessed by CCK-8. **C** Cell proliferation capacity of cells was detected by colony formation experiment. **D** Apoptosis rates were determined by flow cytometry. **E** and **F** Transwell assay revealed the migration and invasion of A549 cells. *P < 0.05, **P < 0.01

To understand the relationship between ARL14 and the malignant phenotype of ARL14, we knocked down ARL14 in A549 cells. According to the RT-qPCR results, the two siRNAs significantly inhibited ARL14 expression, especially ARL14-Sir-1 with higher inhibition efficiency, so it was used for subsequence studies (Fig. 9A). Compared with the cells transfected with si-NC, the cell viability of si-ARL14 transfected A549 cells was significantly suppressed (Fig. 9B). Colony formation assay further confirmed that ARL14 knockdown reduced the number of clones in A549, indicating that it can reduce cell proliferation (Fig. 9C). Besides, flow cytometry indicated that ARL14 knockdown markedly increased the number of apoptotic cells, compared with the si-NC group (Fig. 9D). Furthermore, transwell assay showed that knockdown of ARL14 significantly attenuated the migration and invasion abilities of A549 (Fig. 9E and F). These results suggest that knockdown of ARL14 causes suppression of the malignant phenotype of A549.

## Discussion

Cells can undergo diverse pathways of death, and these pathways is implicated in regulating tumor immune microenvironment as well as tumor metastasis and progression [34, 45], and induction of programmed cell death has emerging as a crucial strategy for cancer therapy [45]. As a form of non-apoptotic cell death, MPTDN attracted more attention in the development of novel cancer therapies [9]. However, the roles of MPTDN in NSCLC is largely unclear. In this setting, we conducted this integrated analysis to uncover the potential roles of MPTDN-related genes in NSCLC.

Among the 39 MPTDN-related genes, 35 genes (90%) were observably dysregulated in NSCLC samples (Fig. 1A), implying the possible roles of MPTDN-related genes in NSCLC. Considering the high heterogeneous property of NSCLC [43], we attempted to investigate the potential of these dysregulated MPTDN-related genes in NSCLC molecular classification, which was the foundation for developing personalized medicine [43]. Two clusters that markedly differed in survival and clinical features were obtained, indicating the successful establishment of MPTDN-related molecular subtypes. This further highlighted the close involvements of MPTDN-related genes in NSCLC. Between these two molecular clusters, 206 DEGs were found, which shared 162 genes with the DEGs screened between NSCLC tumor and control samples. The shared genes were mainly implicated in NK cells mediated cytotoxicity related functions. NK cells are cytotoxic lymphocytes and the primary effector cells against tumors in innate immune system [49]. Thanks to the capacity in enhancing antibody and T cell responses as well as their powerful cytolytic activity towards cancer, diverse NK cell-directed therapies are developed, constituting a main fields of immunotherapy innovation [28, 40]. Currently, NK cell therapy mainly include administration of autologous or allogeneic NK, cytokine supplementation, CAR-NK cells, and monoclonal antibodies [38]. For example, clinical trials have found that pembrolizumab combined with NK cell infusion improves survival in previously treated patients with advanced NSCLC, especially in PD-L1-positive cases [21]. The IL-15 (a factor promoting NK cell proliferation) superagonist ALT-803 in combination with nivolumab exhibited promise against NSCLC in a phase 1b trial [48]. However, insufficient tumor infiltration of NK cells and immune escape of cancer cells from NK cell cytotoxicity limits NK cells-based therapy in NSCLC, and various strategies are proposed to enhance the NK cell infiltration and promote tumor cell susceptibility to NK cell cytotoxicity [32, 44, 52]. The use of NK cells-based foundational therapy with existing immunotherapy/chemotherapy is a promising avenue of exploration.

Three out of the 162 shared genes were identified as prognostic biomarkers, including ARL14, ZDHHC11B, and HLF. Specifically, high expression of ZDHHC11B and HLF were accompanied by better survival times, while those with high expression of ARL14 had worse prognosis. ARL14 is a GTP-binding protein belongs to ADP ribosylation factor family, participating in signal transduction and intracellular transports [6]. Zhang et al. reported that expression of ARL14 was elevated in NSCLC, and its high expression was independently linked to worse survival outcomes of patients with NSCLC [55]. Silencing of ARL14 could restrain the malignant activities of LUAD cells, and induce cell dormancy by blocking ERK/p38 pathway [11]. In this study, we also observed that knockdown of ARL14 markedly inhibited the malignant phenotype of NSCLC cells, including cell viability, proliferation, migration/invasion ability; meanwhile, it also significantly enhanced cell apoptosis rate. ZDHHC11B is a member of zinc finger DHHC proteins (ZDHHC) family, which can regulate the function of a wide range of proteins by their catalyzed protein S-palmitoylation [57]. As a reversible lipid posttranslational modification, protein S-palmitoylation involves in tumorigenesis by affecting important aspects of tumors, such as survival and invasion of tumor cells and anti-tumor immunity [57]. Expression of ZDHHC11B was reduced in LUAD, and such reduced expression predicted poor survival for NSCLC patients [5]. ZDHHC11B up-regulation was observed to significantly inhibit the growth of lung adenocarcinoma in a mouse xenograft model [5]. HLF is a transcription factor belong to PAR BZIP family. In early-relapsed NSCLC tissues, markedly reduced expression of HLF was observed, and its expression was related to early progression and distant metastasis [21]. Similarly, Wang et al. also reported the associations between low HLF expression and worse survival outcomes in LUAD [47]. Overexpression of HLF could restrain lung colonization and distant metastasis in NSCLC [21].

In conducted to facilitate clinical use of these three prognostic biomarkers, a nomogram was established to predict survival for patients with NSCLC. ZDHHC11B seemed to be more weight in the predictive nomogram, followed by ARL14 and HLF. Both calibration curves, DCA and CIC confirmed the accuracy of the predictive nomogram in NSCLC. Besides, we observed a close association between the expression of these three genes and infiltration of various immune cells in NSCLC. For example, ARL14 was negatively correlated with eosinophil, whereas ZDHHC11B and HLF were positively correlated with eosinophil. ARL14 was significantly positively correlated with activated CD4 T cells, while ZDHHC11B and HLF showed opposite results. Eosinophils are rare multifunctional granulocytes, and a key function is the ability to recruit specific immune cells such as CD8 + T cells to tumor sites by releasing various chemokines and cytokines, which are crucial for anti-tumor immune responses [54]. A previous study showed that an increase in peripheral blood eosinophils was considered an early biomarker for improved survival in NSCLC patients treated with nivolumab [31]. Besides, human NSCLC cells, including tumor cell lines and primary tumor cells from clinical patients, can efficiently drive metabolic adaptation of human CD4 + T cells, directing differentiation of regulatory T cells while suppressing effector T cells [46]. Thus, attenuating Treg cells and effectively activating or restoring the effector CD4 + T cell response are important factors for successful tumor clearance [46]. Considering the complexity and versatility of CD4 + T cell subsets, the specific regulatory role of CD4 + T cells in NSCLC needs to be explored in detail in the future. Taken together, these studies further demonstrating the prognostic value of these three genes. In exception to their prognostic values, associations of these three genes with chemotherapy sensitivity was further estimated. Expression of ZDHHC11B was positively associated with the sensitivity of savolitinib, a MET inhibitor, had been recently granted approval in China for treating metastatic NSCLC with MET exon 14-skipping alterations in patients who were unable to tolerate or failed from platinum-based chemotherapy [26]. Expression of both ZDHHC11B and HLF exhibited a significant negative correlation with axitinib, while ARL14 was negatively correlated with the sensitivity of dasatinib and trametinib. All these three drugs used alone or in combination with other drugs have currently investigated in NSCLC by clinical trials [8, 16, 18, 35, 39]. The associations of these three genes with chemotherapy sensitivity contributed to identify the NSCLC patients who might be more likely to benefit from specific chemotherapy drugs. However, a direct link between the expression of these three genes and drug response in NSCLC has not been reported, and further evaluation of the effect of gene expression on drug sensitivity in in vitro or in vivo experiments would make our results more convincing.

To explore the potential biological functions of these three genes in NSCLC, single gene GSEA was conducted. Multiple pathways were dysregulated between NSCLC samples with high and low gene expression. For example, JAK/STAT signaling pathway and toll like receptor signaling pathway were activated in NSCLC samples with high ARL14 expression, and these two pathways were closely implicated in the progression of NSCLC [13, 51, 53]. Furthermore, miRNAs were predicted for these three genes to uncover the possible regulatory mechanism. miRNAs have been closely involved in occurrence and progression of NSCLC by functioning as either regulators, tumor suppressive or oncogenic factors [20]. A variety of miRNAs were predicted to target ARL14 and HLF, implying that these two gene might be potential targets in NSCLC. These findings provided insights to investigate the potential biological functions of these three genes in NSCLC, and should be further confirmed by a series of functional experiments. However, several limitations presented in this study. For example, our sample size was small, and although we used samples from GEO database for external validation, there was a lack of large-scale cohorts to validate our model in depth. In the future, we plan to include a large sample size from multiple centers to improve the generalizability of our results. Moreover, we have only observed the biological function of ARL14 in NSCLC via in vitro experiments, but the specific mechanism needs to be validated in future prospective studies and animal experiments, especially in patient-derived xenograft models [19]. Moreover, we will use a similar approach to explore the role of two other MPTDN biomarkers in NSCLC. As our study continues, addressing these limitations will be key to improving the clinical utility and applicability of our findings in NSCLC treatment.

## Conclusion

In summary, we conducted a comprehensive analysis to understand the multifaceted roles of MPTDN-related genes in NSCLC. Two MPTDN-related molecular subtypes were identified and characterized from prognosis and clinical features. ARL14, ZDHHC11B, and HLF were identified as prognostic biomarkers in NSCLC, which observably correlated with multiple infiltrating immune cells and chemotherapeutics sensitivity. ARL14 may serve a very important target in the treatment of NSCLC.

## Supplementary Information

Supplementary Material 1: Figure S1. Volcano plot and heatmap of DEGs. (A) DEGs between cluster 1 vs. cluster 2. (B) DEGs between tumor vs. control.

Supplementary Material 2: Figure S2. PPI network of 162 overlapped genes.

Supplementary Material 3: miRNA-mRNA regulatory network. Red circle and blue triangles represent the prognostic biomarkers and miRNAs.

## Data Availability

No datasets were generated or analysed during the current study.
