# Supplementary figures and images for: Identification of prognostic biomarker of non-small cell lung cancer based on mitochondrial permeability transition-driven necrosis-related genes and determination of anti-tumor effect of ARL14
Source: Hereditas. 2025 Feb 3;162:16. doi: 10.1186/s41065-025-00379-7 (PMC11789296; doi:10.1186/s41065-025-00379-7)

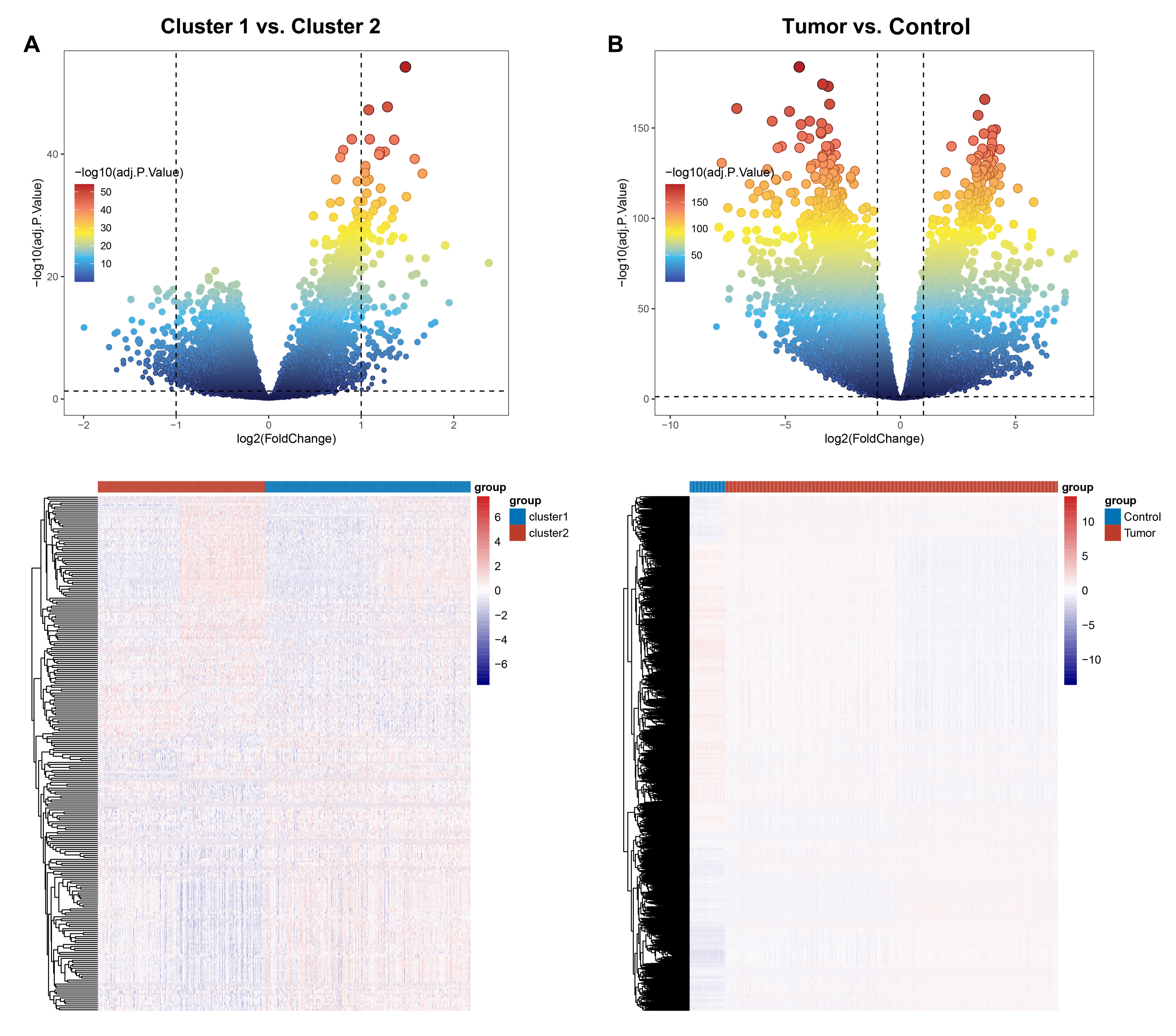

Supplement: Supplementary file 1 — Supplementary Material 1: Figure S1. Volcano plot and heatmap of DEGs. (A) DEGs between cluster 1 vs. cluster 2. (B) DEGs between tumor vs. control. [file 41065_2025_379_MOESM1_ESM.tif]

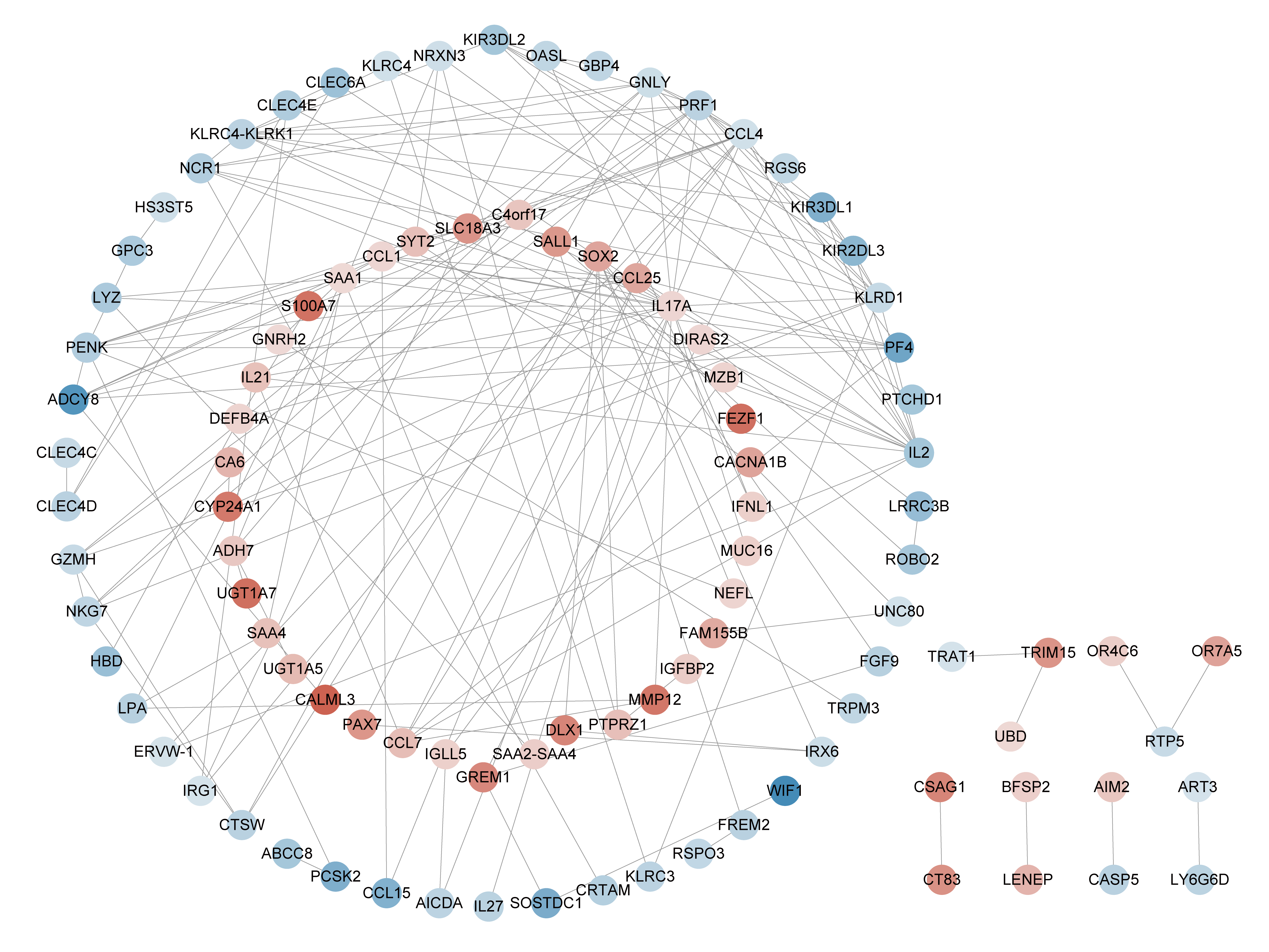

Supplement: Supplementary file 2 — Supplementary Material 2: Figure S2. PPI network of 162 overlapped genes. [file 41065_2025_379_MOESM2_ESM.tif]

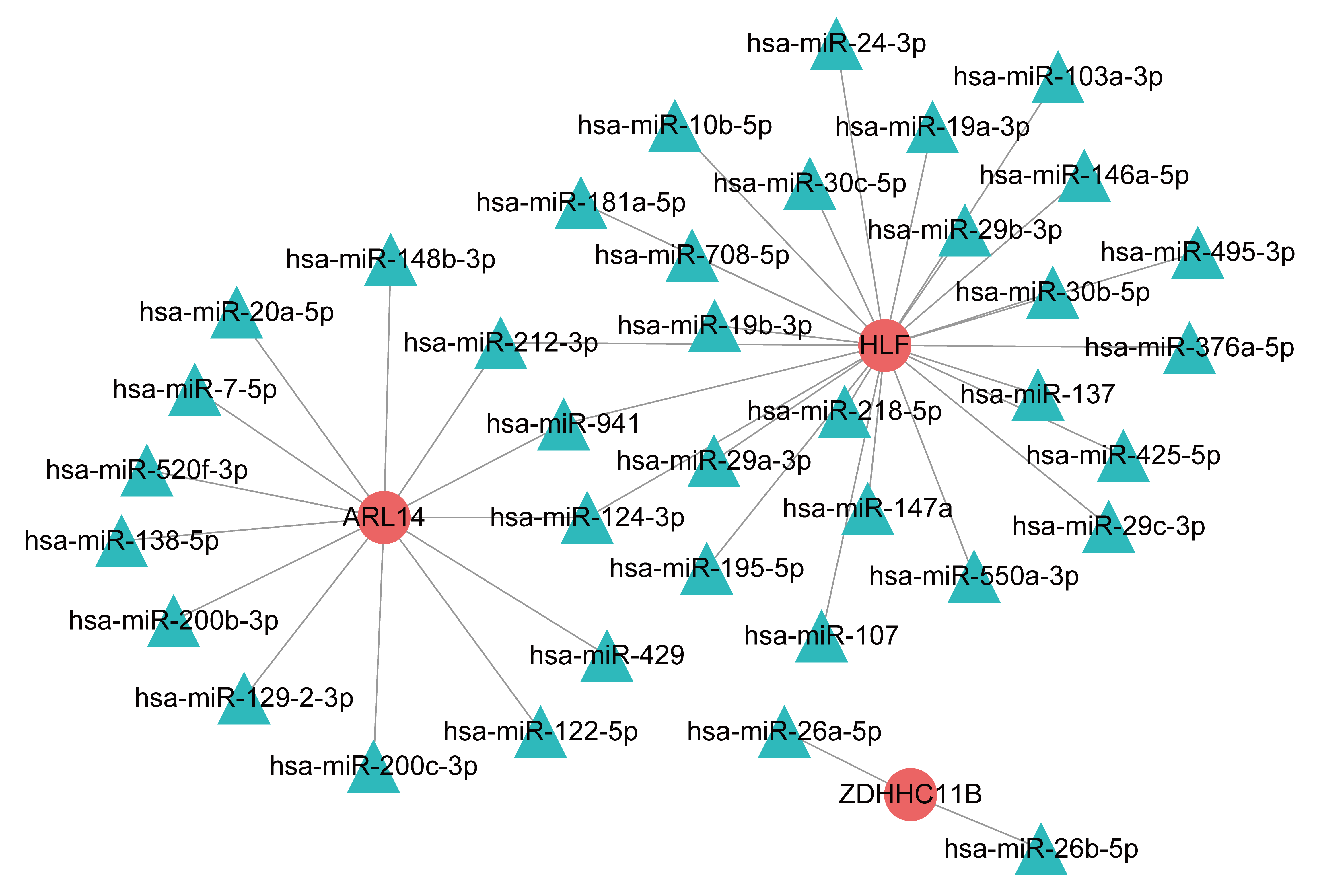

Supplement: Supplementary file 3 — Supplementary Material 3: miRNA-mRNA regulatory network. Red circle and blue triangles represent the prognostic biomarkers and miRNAs. [file 41065_2025_379_MOESM3_ESM.tif]
